# Industrial applications of 3D printing to scale-up production of COVID-19-related medical equipment

**DOI:** 10.2217/3dp-2021-0003

**Published:** 2021-08-09

**Authors:** Muhammad Zaheer Abbas

**Affiliations:** 1 Faculty of Business and Law, Queensland University of Technology, Brisbane, Australia

**Keywords:** 3D printing, additive manufacturing, COVID-19, health emergency, industrial application, medical equipment

## Abstract

Additive manufacturing or 3D printing allows the rapid conversion of information from digital 3D models into physical objects. The current COVID-19 crisis underscored the value of 3D-printing technology in addressing critical shortages in the medical product supply chain. This article provides a review of the significant role of additive manufacturing technologies in addressing the COVID-19 situation. This article concludes that 3D printing has an important role in global public health because of its potential to adapt to emerging situations far more easily and quickly as compared with conventional manufacturing methods. There is a need for further research to improve the technology to mass produce better quality products more economically. Currently, the 3D-printing industry is concentrated in the US and Western Europe. Policy efforts are needed to tap all markets across the globe in order to be better prepared for a future pandemic.

The COVID-19 pandemic has ravaged the world since the end of 2019. There is a hierarchy of medical technologies in terms of their utility to combat the COVID-19 crisis. Vaccines lie at the top of the hierarchy to curb the spread of the pandemic. Understandably, the focus of international cooperation and collaboration has been on developing safe and effective vaccines as soon as possible. Diagnostics and therapeutics can be placed second in the hierarchy. Then come personal protective equipment (PPE) and other medical equipment. Although low in the hierarchy, PPEs are critically needed as the health and safety of healthcare professionals, who face heightened risks of infection, remain threatened without proper protection. Healthcare professionals comprised 21% of cases in the 2002 severe acute respiratory syndrome outbreak [1]. To prevent such an alarming trend and to limit the devastating health and socio-economic impact of the COVID-19 pandemic, it is essential to properly support and safeguard frontline healthcare workers.

The COVID-19 health crisis put global healthcare systems under critical strain. Vulnerabilities of conventional supply-chain mechanisms were exposed as there was a significant shortage of PPE and other materials for medical personnel as well as for patients and regular people [2]. Plagued with a serious lack of resources, healthcare providers had to seek alternative sources of critically needed medical equipment. The global crisis increased the visibility of the possibilities provided by 3D printing and triggered heightened attention and urgent interest in its extraordinary potential. Although 3D printing does not offer much help in the quest for COVID-19 vaccines, it has a high level of utility in diagnostic testing, PPE supplies and other medical equipment supplies. It provides the much-needed flexibility in the manufacturing and supply chains [3].

Additive manufacturing is defined as the ‘process of joining materials to make parts from 3D-model data, usually layer upon layer, as opposed to subtractive manufacturing and formative manufacturing methodologies’[4]. 3D printing is ‘fabrication of objects through the deposition of a material using a print head, nozzle or another printer technology’ [4]. This method of manufacturing is ‘in contrast with conventional manufacturing processes in which physical shapes emerge either by removing material, as in machining, or changing the shape of a set volume of material’ [5]. Each of these successive layers of raw material ‘can be seen as a thinly sliced horizontal cross-section of the eventual object’ [6]. Unlike any other manufacturing technology, this advanced fabrication method manufactures 3D tangible products from a predesigned computer-driven 2D blueprint or digital model, called a computer-aided design (CAD) file, of the required shape [7]. This unique and versatile manufacturing method suits time-sensitive innovation and manufacturing as it does away with the time-consuming and costly tooling and machining requirements.

The current health emergency put a fresh light on the unique capabilities of 3D printing to provide innovative solutions to emerging problems and to address medical product supply chain disruptions in an emergency. As noted by M Attaran, “the strengths of 3D printing are that it can be anywhere, can print virtually anything, and adapt on the fly” [8]. These features help 3D printing to fill the critical gaps in the supply chain of critically needed medical equipment. This article provides a review of the significant role of additive manufacturing technologies in addressing the COVID-19 situation. This review, in respect of fast-moving developments, draws upon a wide range of sources including peer-reviewed publications, blogs, quotations from stakeholders, media reports and real-world examples. This review is important as it is specifically focused on the industrial applications of 3D printing in response to the COVID crisis. This article will benefit the field and patients in the future as it draws attention of policymakers to key policy concerns and makes recommendations for policy initiatives to make better use of 3D-printing technology in a future pandemic. It offers a speculative and forward-looking perspective of the role of 3D printing in a future health emergency.

This article has a three-part structure including the introduction and the conclusion. Part II considers the COVID-19-related industrial applications of 3D printing. In particular, it explores the applications of 3D printing to fabricate PPE (like face masks, face shields), medical and testing devices (like ventilator splitters and nasal swabs), and useful accessories (like hands-free door openers). The conclusion in Part III calls for improvements in 3D-printing technology to make it the fully-fledged first-choice manufacturing technique for mass production of medical equipment in response to a future pandemic. It also calls for a geographically dispersed growth of the 3D-printing industry so that a wide range of the global population can benefit from its unique benefits.

## Industrial applications of 3D printing in response to COVID-19

The 3D-printing industry is better equipped with capabilities to respond to a health emergency like COVID-19. When there is a need to innovate and manufacture products under extraordinary time pressure, 3D printing has the potential to rise to the challenge as it enables an agile manufacturing environment to scale production speedily. Traditional manufactures cannot respond quickly because the turnaround time is very high. As compared with conventional numerically-controlled cutting machines, 3D-printing technology makes one-offs more easily and quickly [9]. This technology makes it possible to robustly validate an idea, then a design, then iterations, and finally to test functionalities of the printed product [10]. A product can rapidly go from an idea to design, to prototype and manufacturing in a single day [11]. Once a physical product is fabricated, if something is not working or some additional feature needs to be added, one can quickly make alterations in the digital model to print a modified physical object. 3D printing not only makes it easier and cheaper to build prototypes but also decreases the time between iterations [12]. These important advantages contribute to both time- and cost-efficiency of 3D printing. As noted by Lucas Osborn:

Without this technology, individuals might have needed to hire a draftsperson to create technical drawings. They also would have needed to take those drawings to a manufacturing intermediary who would facilitate prototype construction. Depending on the invention, prototyping might have required expensive tooling or handcrafting and might have taken weeks to complete. Instead, 3D-printing technology dramatically lowers the costs (and time) of prototyping [13].

The ability to do multiple iterations, with low lead times, is important as there can be several possibilities in a designer’s mind to address a particular problem. This ability to design anything quickly and print right away is unique to 3D-printing technology. For instance, to reduce the risk of infection from door handles, the Belgian Company materialized a screw-free door opener extension within 4 h [14]. The extension makes it possible to open a door with a forearm instead of using hands. In traditional manufacturing, which has a much longer product development cycle, the designers have to cross their fingers and see how things work out. A traditional manufacturer cannot adapt quickly under extraordinary time pressure in a pandemic situation. The product development process can take days and even weeks for traditional manufacturers [11]. This is a major drawback, especially in situations when there a need to cover the time-sensitive high demand for critical products.

Moreover, the ease of achieving customizability in shape distinguishes 3D-printing technology from traditional manufacturing methods. 3D-printing’s geometrical freedom allows adaptability and flexibility in problem-solving. Mass customization and low-volume manufacturing allow the making of more relevant products without investing excessive time and resources [15]. The customization feature becomes even more profound when 3D printing is paired with 3D scanning. 3D scanners can be used to quickly turn a physical object into a digital object [13]. 3D digitizing is the ‘method of acquiring the shape and size of an object as a 3D representation by recording x, y, z coordinates on the object’s surface and through software the collection of points is converted into digital data’ [4]. The digital design file or CAD file can be simply iterated to make any alteration or adjustment. On the contrary, making customized products by deploying traditional manufacturing techniques is both costly and time-consuming as design modifications cannot be easily achieved [10]. A traditional manufacturer may need to make costly changes to the manufacturing process, requiring new tools and molds, to realize a change in design. Even with additional costs and efforts, conventional techniques are constrained in achieving optimal customizability because reaching some places of the geometry is simply beyond the capacity of traditional manufacturing methods which offer the possibility of making much less versatile objects within a limited spectrum of shapes. Computer-aided designing has, “advanced our capacity to understand and visualize mathematical forms that extend beyond the limits of traditional Euclidean geometry” [16]. 3D printing allows us to conveniently build those complex forms.

Complexity is free in 3D printing because complex designs can be made with little or no additional cost [17]. Complexity costs the same as simplicity as a 3D printer can print self-assembled complex products without involving any additional efforts or arrangements. The zero-marginal cost of manufacturing complexity, with no constraints on the geometry, is significant in enriching approaches to problem-solving, especially in an emergency, because a lot of new possibilities emerge when complexity is free and easily achievable. The opening of new possibilities is particularly important for personalized applications in the medical field since every patient’s and caregiver’s geometry is different [18]. On the contrary, making complex products by employing traditional manufacturing techniques is both costly and cumbersome [19]. Extra complexity requires a more expensive mold with more parts [20]. Multiple parts of complex products need to be designed and manufactured separately and then assembled manually, often involving human effort. The requirement of postproduction assembly also adds to labor costs.

Furthermore, combining different raw materials into a single product is difficult in traditional manufacturing methods. Customizability in the composition is much easier to achieve while 3D-printing products. There are continual advances in combining antimicrobial polymers with 3D-printing materials to improve their antimicrobial activity against viruses, fungi and bacteria [21]. Materials like copper oxide, titanium oxide, zinc oxide, magnesium oxide have shown biocidal effects for a wide range of viruses [21,22]. The possibility of fabricating products by employing antimicrobial filament creates opportunities for promising applications of 3D printing in response to the COVID-19 pandemic. For instance, the addition of copper oxide into protective face masks can result in achieving effective anti-influenza properties and reducing the viral load on the masks [22]. The high cost of antimicrobial filaments can be a concern. Low-cost options, like N-halamines, can be considered for incorporation into polymers [23].

This section discusses some of the most notable industrial applications of 3D printing during the current health crisis. It also proposes improvements in the current capabilities of 3D printing in order to be better prepared for a future health emergency.

### Personal protective equipment

PPE is key to the safety of both the patient and the caregiver given the extreme contagiousness of the COVID-19 virus and the volume of infected patients. According to the WHO guidelines, proper PPE is necessary for all medical services in hospitals and other healthcare settings [24]. Shortages of PPE, resulting from supply chain disruptions and demand increases, put healthcare professionals in grave danger. The 3D-printing community stepped into the void to offer support to hospitals, nursing homes and even refugee camps [25]. The 3D-printing industry fabricated various PPE devices during this COVID-19 pandemic.

#### Protective face masks

**Figure 1.**
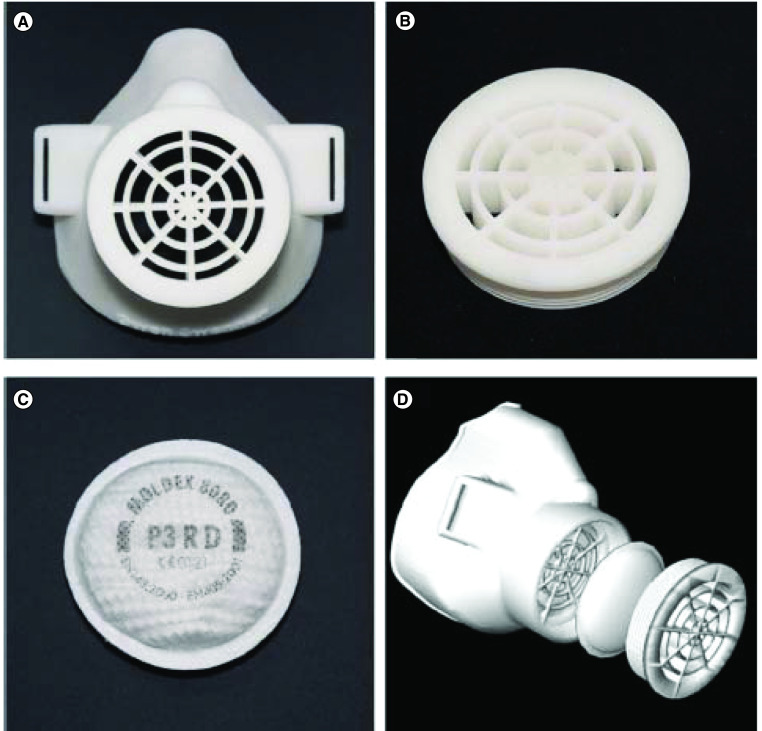
Individualized custom-made 3D-printed protective face mask. Reprinted with permission from [26] © Elsevier (2020).

Protective face masks, as shown in Figure 1, have an important role in slowing the spread of the COVID-19 virus, which can be spread through respiratory droplets. Protective face masks are critical not only for medical personnel who treat infected patients but also for the general public professionals who test, transport or engage with virus victims in any way.

3D printing has a high potential to be used for customized and personalized applications in the medical field [27]. The flexibility of 3D printing to fabricate customized objects of any desired shape – without requiring much time, effort and monetary cost – comes handy in producing personalized face-seal designs. A generic face mask might not match with a human face due to variation in shape and size because the face and nose lengths, chin arc, jawline and nose protrusion measurements vary from person to person [28]. Small particles can possibly enter the wearer’s breathing zone due to the loose fit between the wearer’s face and the surface of the generic mask [22]. 3D-printing technology enables personalizing of face masks to achieve a better fit on an individual basis and improved functionality in blocking contaminated air from entering around the edges of the mask [29]. Personalized face masks significantly improve comfort by reducing and evenly distributing the contact pressure [30]. Comfort refers both to the comfort of the breathing process and the comfort of the wearer’s face. As detailed in Table 1, there were significant industrial applications of 3D printing to manufacture face masks during the current health crisis.

**Table 1.** Notable 3D-printing companies manufacturing face masks in response to the COVID-19 crisis.

Company	Country
Materialise	Belgium
AddiFabb	Denmark
Trinkle	Germany
Forecast 3D	USA
3D Systems	UK
Essentium	USA
Nexteer	USA
MatterHackers	USA
Flowbuilt Manufacturing	USA
Evonik	Germany
Ferrovial	Spain
Fathom	USA
Fortify	USA
Voodoo	USA
Caracol-AM	Italy
Nagami Design	Spain

Data taken from [31,32].

#### Protective face shields

**Figure 2.**
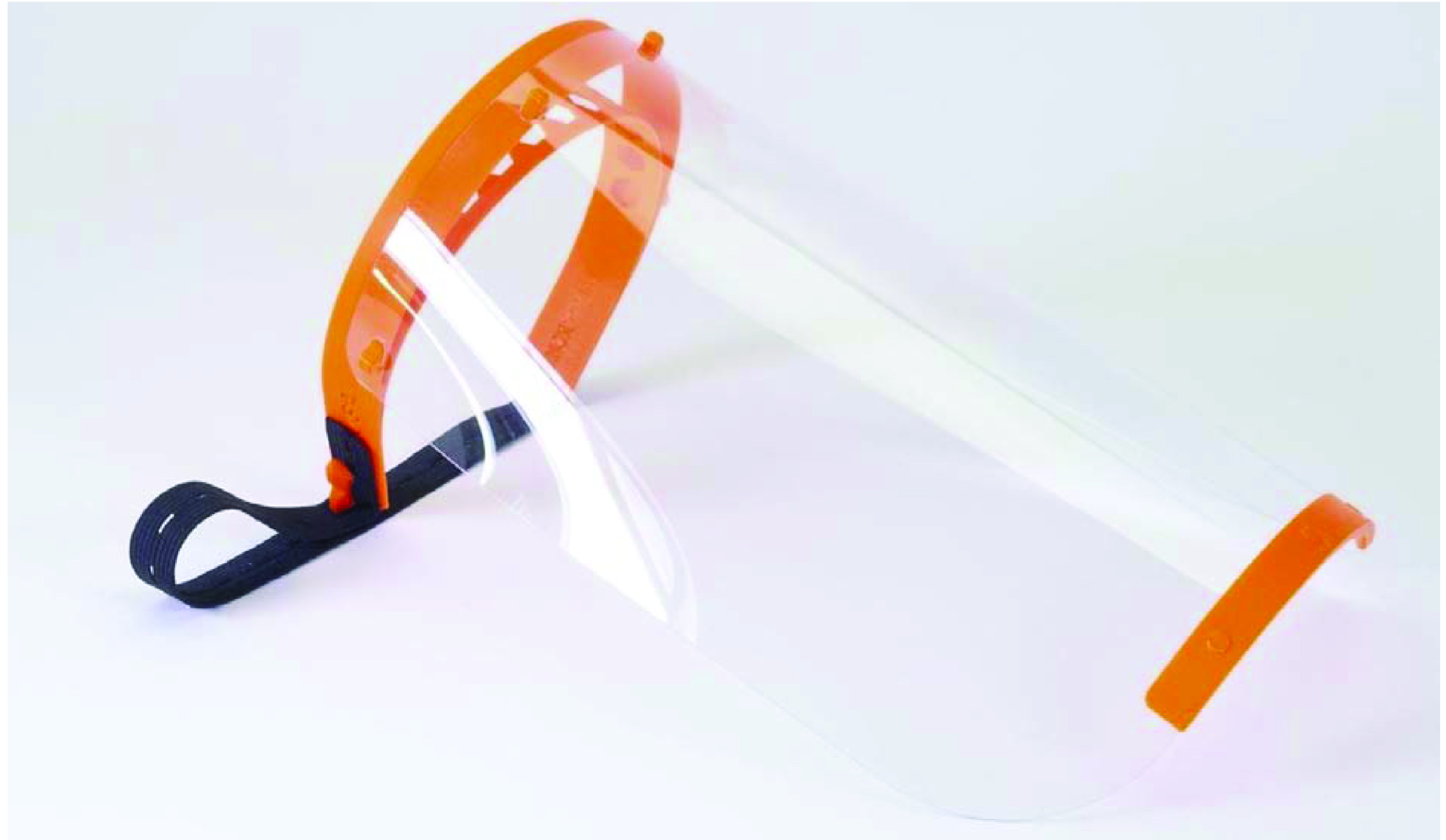
3D-printed protective face shield. Reproduced with permission from [29], licensed with CC BY 4.0.

Protective face shields, as shown in Figure 2, have threefold importance. First, face shields protect healthcare professionals from respiratory droplets resulting from sneezing or coughing or splash of any respiratory secretion from COVID-19 patients [33]. Second, face shields help preserve the longevity of face masks by preventing their contamination [34]. This capability of face shields to mitigate the soiling of face masks is particularly important in a pandemic situation when acute shortages in supplies of face masks are caused by disruptions and high demand. Third, face shields help slow down the spread of the COVID-19 virus as they prevent touching of face, nose and eyes [34].

The face shield is created by 3D printing the solid headband and chin piece and attaching a separately sourced transparent plastic sheet or faceguard [29]. The cost of fabricating a face shield, by using this straightforward method involving minimal assemblage requirement, ranges from $10 to $177 depending on the durability and the printing materials used [35]. Maintenance of 3D face shields is easy and cost effective as the defective parts can easily be removed and replaced in the case of damage [35]. It is essential to clean and disinfect all parts of a 3D-printed face shield before using it. As detailed in Table 2, there were significant industrial applications of 3D printing to manufacture face shields during the current health crisis.

**Table 2.** Notable 3D-printing companies manufacturing face shields in response to the COVID-19 crisis.

Company	Country
Issinova	Italy
NGen	Canada
Fast Radius	USA
Prusa3D	Czech Republic
BCN3D	Spain
Titan Robotics	USA
Prodways	France
Forecast 3D	USA
Carbon	USA
Blue Origin	USA
Nexteer	USA
Paragon	UK
Azul 3D	USA
Fathom	USA
SmileDirectClub	Canada

Data taken from [31].

### Medical & testing devices

#### Ventilator splitters

A ventilation system is the last hope for critically ill COVID-19 patients facing respiratory failure. A ventilator, typically found in intensive care units is, "a medical device used to assist patient breathing (by mechanical or pressure control) by moving breathable air into and out of the lungs with intubation” [35]. By providing positive pressure to the lungs, it supports a critically-ill patient’s respiration by maintaining an adequate level of oxygen concentration in the arterial blood [36].

The supply of available ventilators was too low to cater to all COVID-19 patients with acute respiratory distress who needed invasive mechanical ventilation [29]. Expanded use of ventilators was made possible by using 3D-printed ventilator splitters [6]. By making this quick and feasible adjustment, the capacity of a single ventilator can be increased to ventilate up to four simulated adults for a limited time [22]. These specially designed splitters divide the airway of the ventilator [37]. 3D-printing technology enables the customization of designs of these splitters to minimize dead-space volume and prevent air leakage [22]. Table 3 enlists some of the 3D companies that manufactured ventilator splitters during the current health emergency.

**Table 3.** Notable 3D-printing companies manufacturing ventilator splitters in response to the COVID-19 crisis.

Company	Country
3D Systems	UK
Protolabs	France
Evonik	Germany
EnvisionTEC	USA
Fathom	USA
CPR Technology	Italy
Roboze	Italy
Photocentric	UK
Proto21	Dubai

Data taken from [31].

#### Nasal swabs

**Figure 3.**
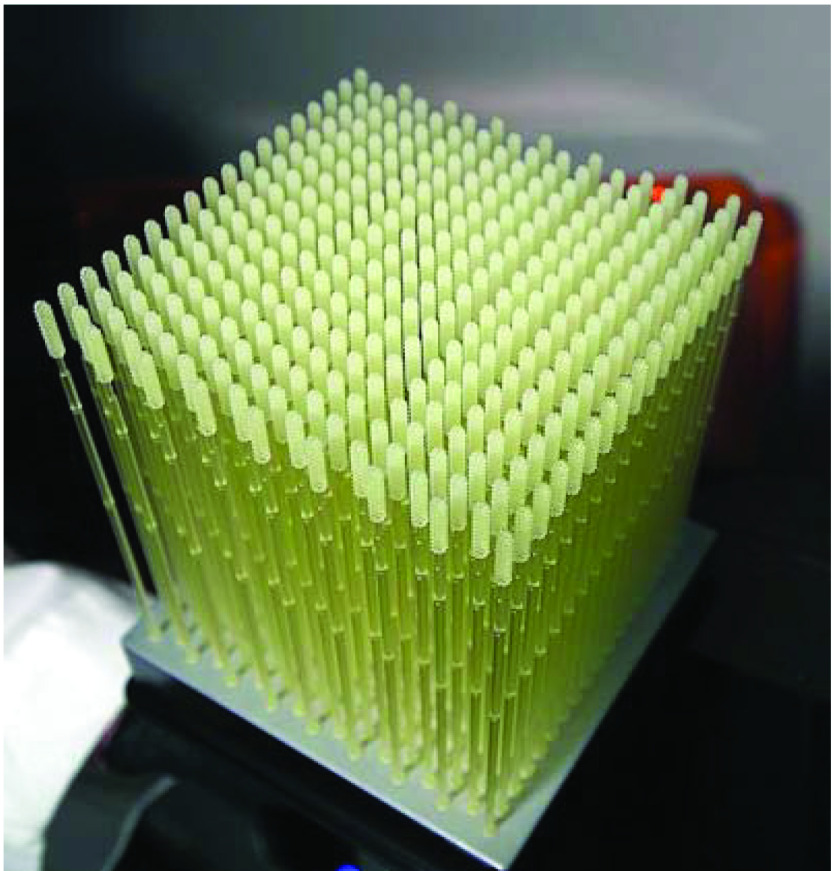
3D-printed nasal swabs. Photo courtesy of USF Health [41].

Diagnostic testing is crucial to identify and isolate known cases and to assess risks of further spreading of COVID-19. As noted by TA Ghebreyesus, WHO Director-General, “You cannot fight a fire blindfolded. And we cannot stop this pandemic if we don’t know who is infected. We have a simple message for all countries: test, test, test. Test every suspected case” [38]. The rapid spread of COVID-19 resulted in acute shortages of appropriate nasopharyngeal swabs [39] or nasal swabs, a medical device roughly 15 cm in length and 2 to 3 mm in diameter designed to collect secretions from the posterior nasopharynx for diagnostic sampling (Figure 3) [40]. The severe shortages were caused by both decreased supply resulting from manufacturing stoppages and unprecedented demand [39].

The large-scale testing desperately required an adequate supply of nasal swabs, which provide the highest sensitivity for diagnosing COVID-19 infection [39]. There were severe challenges in ramping up testing across the globe because the conventional manufacturing of nasal swabs was centralized in Italy [3]. Moreover, the conventional method of manufacturing diagnostic swabs is tedious and limited. Industrial use of 3D-printing technology helped to promptly address shortages for COVID-19 testing [42]. With a total print time of around 3 h and 40 min, 300 nasal swabs can be 3D printed at once [43]. The cost of 3D printing the swabs is around USD 0.25 per swab [29]. Table 4 enlists some of the 3D companies that manufactured nasal swabs during the current health emergency.

**Table 4.** Notable 3D-printing companies manufacturing nasal swabs in response to the COVID-19 crisis.

Company	Country
Markforged	USA
Forecast 3D	USA
Carbon	USA
Paragon	UK
EnvisionTEC	USA
Formlabs	USA
Origin	USA
Structo	Singapore

Data taken from [31].

### Door handle accessories

**Figure 4.**
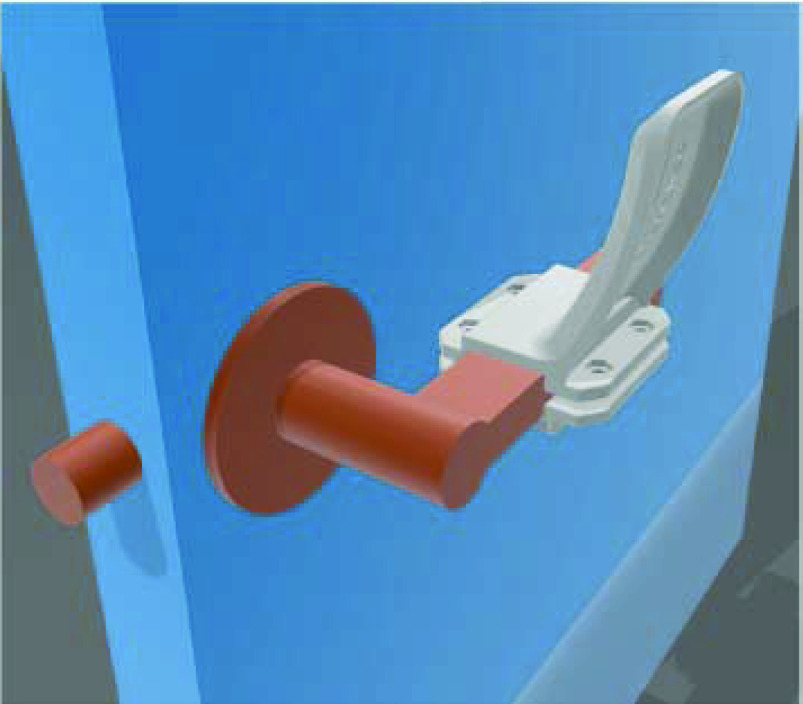
3D-printable model of door handle accessory. Reproduced with permission from [29].

Faced with the risk of highly-transmittable viral infection, hospitals are bound to take meticulous precautionary measures to limit the spread of the COVID-19 virus via direct contact. Door handles in hospitals are one of the most common mediums for infection as they "may act as vectors for the transmission of viruses and multidrug-resistant organizations responsible for nosocomial infections" [44]. For the sake of ward control and patient privacy, hospitals normally have a large number of doors. Regular surface cleaning does not completely address the risk of transmission from door handles which are routinely subjected to a lot of physical contact [29]. The use of automatic doors may be considered to avoid the risk of spreading infection from door handles, but most hospitals may lack financial resources to materialize this option. The same goal of avoiding direct skin-to-surface contact can be achieved in a much more economical way by using 3D-printing technology to design and manufacture hands-free door openers. This novel accessory creates an extension to existing door handles to enable opening doors by using forearms instead of hands (Figure 4). Operating doors with forearms substantially reduces the risk of transmitting the COVID-19 virus because this body part is rarely used to touch the mouth, nose and eyes [45].

Even the simplest interventions to limit transmission can play a significant role in slowing down the spread of the COVID-19 virus. Ready-to-print designs of door handle accessories are available for free download and fabrication by using a variety of 3D-printing approaches and materials [46]. 3D-printed door handle accessories are easy to install and do not require drilling holes. To assist hospitals in setting it up, Materialise provided video instructions on “How it works” [47]. Table 5 enlists some of the 3D companies that manufactured door handle accessories during the current health emergency.

**Table 5.** 3D-printing companies manufacturing door handle accessories in response to the COVID-19 crisis.

Company	Country
Sintratec	Switzerland
Materialise	Belgium
Creatz3D	Singapore

Data taken from [31].

There were also some unanticipated contributions from the industry that deserve a mention in this section (Table 6). Many automobiles manufacturers volunteered their 3D-printing facilities for the fabrication of COVID-19-related medical products [8]. 3D printing allows companies the flexibility to adapt quickly and convert their production in the light of changing needs of industry or society. This unique capability of 3D printing empowers companies to practically contribute physical objects in response to an emergency. Several unanticipated contributions from the industry were possible only because of the enabling role of 3D printing. No other manufacturing method allows such a quick adaptation to deliver in an entirely new and irrelevant field. As noted by a spokesperson for Volkswagen, “Medical equipment is a new field for us. But as soon as we understand the requirements, and receive a blueprint, we can get started” [48]. Likewise, a spokesperson for BMW said, “the production of components using 3D-printing technology is a possibility” [48].

**Table 6.** Notable unanticipated contributors of 3D-printed medical equipment in response to the COVID-19 crisis.

Company	Category	Notable 3D-printing contributions
Apple	Technology company	Face shields, face masks
Blue Origin	Technology company	Face shields
HP Inc.	Technology company	Nasal swabs, face masks, hands-free door openers
Tesla	Electric vehicle and clean energy company	Ventilator valves
Ford	Automobiles manufacturer	Ventilator valves
Volkswagen	Automobiles manufacturer	Face shields, face masks
Nissan	Automobiles manufacturer	Face shields
Ferrari	Automobiles manufacturer	Ventilator valves, face masks
General Motors	Automobiles manufacturer	Ventilator valves, face shields, face masks
Honda	Automobiles manufacturer	Face shields
Holden	Automobiles manufacturer	Ventilator valves
Airbus	Aerospace manufacturer	Hospital visor frames
Boeing	Aerospace manufacturer	Face shields, face masks
SpaceX	Aerospace manufacturer	Ventilator valves
Goodyear	Tire manufacturer	Face shields

Data taken from [14,45,49].

3D printing creates possibilities that no other manufacturing method can potentially create. Without the power of 3D printing, one could hardly imagine that technology companies, manufacturers of automobiles, aerospace corporations, and tire manufacturing companies would ever be able to deliver medical equipment in a health emergency.

Despite its unique capabilities and remarkable contributions during the COVID-19 crisis, at present, 3D printing is aptly suitable only for low-volume and specialized products. When it comes to large-scale production, 3D printing does not compete with traditional production processes, like injection molding. Desktop 3D printers, based on stereolithography or fused deposition modeling technology, have been most commonly used for 3D printing medical devices [50]. These printers are not fast as they “rely on stepwise, layer-by-layer approach to fabrication of objects, which inevitably takes time and is the main factor limiting manufacturing throughput” [51]. The fabricating time increases even more if there is a demand for a higher-quality product [25]. For the widespread adoption of 3D printing as a mainstream manufacturing technique, it is important to tackle the problem of slow printing speed.

3D printing, in its current form, is not a viable alternative to factory production also because of the cost of mass production. Although manufacturing of parts in metal is possible using 3D printers, the process would be far more expensive as compared with traditional factory production [52]. The cost per item is high if identical copies are 3D manufactured at scale because the base material is costly [10]. To provide a more robust production alternative, the next goal of 3D-printing technology should be to provide mass-production capabilities with a key focus on reducing cost, improving accuracy, and enhancing the speed and reliability of 3D printers. In order to make 3D printing the fully-fledged first-choice manufacturing technique for mass production of medical equipment in response to the next pandemic, significant research and concerted policy efforts are needed in all the different additive manufacturing processes – like binder jetting, directed energy deposition, material extrusion, material jetting, powder bed fusion and others.

Moreover, quality control and compliance with health standards can be a concern. To ensure safety and efficacy, a certain level of quality control is needed before a 3D-printed medical device reaches the general market. Compared with advanced level equipment, like biomedical devices and tissue-engineered scaffolds, 3D-printed medical products manufactured in response to COVID-19 are less complicated and may require less stringent regulatory and dimensional accuracy standards [10]. A lower degree of risk in the intended use of a device is expected to require lower level of regulatory control. Some level of testing and validation is, however, required in normal circumstances to make sure that 3D-printed medical devices do not cause any unintentional and avoidable harm to the patients.

In the COVID-19 emergency, leading to life-or-death choices due to supply shortages, it was not possible for many governments to carry out formal regulatory testing by using standardized methods and enforce strict regulatory controls on 3D printing, which provides a sustainable backup solution to address supply chain failures. For safety of patients and healthcare workers, it is important to follow a certain level of safety standards and quality measures even in an emergency situation. Learning from the COVID-19 experience, governments may develop safety protocols related to the use of 3D printing in a future health emergency. To maintain the acceptable quality of health-related 3D-printed objects, these protocols should provide guidelines about CAD modeling of medical devices, the suitable 3D-printing technology and appropriate materials to be used to fabricate such devices [53].

It is important to provide a framework for additive manufacturing of medical devices because printing parameters, fabrication materials, fiber thickness and printing raster orientation have an impact on the quality and mechanical properties of 3D-printed parts [54]. To address the dangers of using 3D printing in a health emergency, concerning regulative authorities of governments should develop mechanisms for expedited evaluation and approval/certification of prototypes of urgently-needed medical equipment. Approvals and certifications are very complicated in the absence of clearly defined standards [55]. Government authorities should also issue guidance documents to make designers and manufacturers of 3D-printed medical parts aware of their potential legal liability. Concerted policy and regulatory efforts are needed to realize the goal of using 3D-printing technology to swiftly fabricate safe and certified medical products on a scale in a future pandemic.

## Conclusion

3D printing is not yet another manufacturing technique, it is a completely different technique. Because of its unique capabilities, 3D printing has a major role in health emergencies like COVID-19. It offers the much-needed flexibility, agility and diversity of solutions in response to an emergency. 3D printing is a resilient technology as it delivered the much-needed medical supplies in a timely fashion under extraordinary time pressure, despite all odds that negatively impacted conventional supply chains during the pandemic. It provides sustainable possibilities in ways that cannot be even imagined by traditional manufacturers. The flexibility of this technology to design and fabricate customized objects – without requiring much time, effort and monetary cost – comes handy in an emergency like the COVID-19 pandemic.

It can be noted from the above analysis that industrial 3D printing had a significant impact in addressing COVID-19 related shortages of materials. Despite its unique capabilities and remarkable contributions, industrial 3D printing does not compete with traditional production processes in speed, cost and quality when it comes to mass production. Significant research and concerted policy efforts are needed in all the different 3D-printing processes in order to make it the fully-fledged first-choice manufacturing technique for mass production of medical equipment in response to a future pandemic.

More importantly, it can be noted that the 3D-printing industry mostly concentrated in the USA and Western Europe manufactured COVID-19-related medical materials. There are no examples of 3D manufacturing in response to COVID-19 from markets in low- and middle-income countries. It is a cause of serious concern if a vast majority of markets in the developing world remain untapped. Affordability and availability of medical technologies are major concerns in low- and middle-income countries. The 3D-printing industry can improve the quality of healthcare in these countries by addressing these concerns in an inexpensive way. Policy efforts need to be made on a priority basis to tap all markets across the globe in order to be better prepared for a future pandemic.

## Future perspective

The combination of human creativity and 3D-printing technology can solve critical problems in an emergency situation. Shared objectives can be accomplished more quickly if creative people from a wide range of professional backgrounds are familiar with the capabilities of 3D printing. To open up new possibilities in health-related applications of 3D printing, it would be desirable to create an environment where medical professionals and engineers can work together to solve emerging problems. In the foreseeable future, governments may be expected to support education and training in respect of 3D printing. As well as including 3D printing in the curriculum at the primary school level, the disciplines may be taught in the engineering and medical curriculum right from the undergraduate level.

Learning from the COVID-19 experience, governments may be expected to support decentralization of manufacturing capabilities and diversification of supply chains. It will improve responsiveness and product availability while reducing transportation costs and risks associated with cross-country transportation. Most of the hospitals and medical centers, at least in economically-advanced countries, may be equipped with their own in-house 3D-printing capabilities to produce patient-specific medical parts and to break their dependence on global supply chains. Moreover, governments may be expected to remove regulatory uncertainties regarding the use of 3D printing in the medical sector. A clear regulatory framework and a specialized mechanism for fast-track safety review and quality validation of CAD designs may be provided to use the full potential of 3D printing as a life-saving technology in a future pandemic.

More importantly, the overall cost of 3D printing is expected to decline further in the future. Development of more versatile 3D printers can be foreseen that can fabricate better quality parts more quickly and cost effectively. Greater resolution and manufacturing precision will be achieved at lower costs and faster rates. The breadth of 3D-printing applications will continue to grow. New technologies related to 3D printing will emerge and the scope of printing materials will expand with the passage of time. 3D-printing technology will become more accessible in economically advanced countries. It will shape the future of many industries and new players will enter the market. However, concerted efforts would be required at global and national levels to promote 3D printers to low- and middle-income countries.

**Executive summary**

**Introduction**

- The importance of personal protective equipment (PPE) for the health and safety of healthcare professionals is highlighted.
- The concept of additive manufacturing is explained in the context of COVID-19 and supply-chain disruptions.

**Industrial applications of 3D printing in response to COVID-19**

- The unique capabilities of 3D-printing technology are emphasized.
- 3D-printing technology rose to the challenge by enabling an agile manufacturing environment to scale production speedily.
- 3D-printing technology’s unique ability to do multiple iterations, with low lead times, is important in an emergency situation to fabricate time-critical medical products.
- Making customized products by deploying traditional manufacturing techniques is both costly and time-consuming.

**Protective face masks**

- 3D-printing technology enables personalizing of face masks to achieve a better fit on an individual basis.
- Personalized face masks significantly improve comfort by reducing and evenly distributing the contact pressure.

**Protective face shields**

- Protective face shields are important in protecting healthcare professionals from respiratory droplets, preserving the longevity of face masks, and preventing contact with face, nose, and eyes.
- The cost of 3D printing face shields is low and maintenance of such shields is easy in the case of damage.

**Ventilator splitters**

- A ventilator helps in maintaining an adequate level of oxygen concentration in the arterial blood by providing positive pressure to the lungs.
- The available supplies of ventilators were too low to cater to unprecedented demand for respiratory support apparatus during the current COVID-19 crisis.
- Expanded use of ventilators was made possible by using 3D-printed ventilator splitters.

**Nasal swabs**

- The rapid spread of COVID-19 resulted in acute shortages of appropriate nasopharyngeal swabs.
- Industrial use of 3D-printing technology helped to promptly address shortages for COVID-19 testing.

**Door handle accessories**

- Door handles in public places are routinely subjected to a lot of physical contact.
- The goal of avoiding direct skin-to-surface contact was achieved in an economical way by using 3D-printing technology to design and manufacture hands-free door openers.

**Regulatory compliance**

- In the current health emergency, it was not possible for many governments to carry out formal regulatory testing and enforce strict regulatory controls on 3D printing.
- Learning from the COVID-19 experience, governments may develop protocols related to the use of 3D printing in a future health emergency.
- Concerning regulative authorities of governments should develop mechanisms for expedited evaluation and approval/certification of prototypes of urgently-needed medical equipment.
